# Impact of Exogenous Application of Potato Virus Y-Specific dsRNA on RNA Interference, Pattern-Triggered Immunity and Poly(ADP-ribose) Metabolism

**DOI:** 10.3390/ijms23147915

**Published:** 2022-07-18

**Authors:** Viktoriya O. Samarskaya, Nadezhda Spechenkova, Nikolay Markin, Tatyana P. Suprunova, Sergey K. Zavriev, Andrew J. Love, Natalia O. Kalinina, Michael Taliansky

**Affiliations:** 1 Shemyakin-Ovchinnikov Institute of Bioorganic Chemistry of the Russian Academy of Sciences, 117997 Moscow, Russia; viktoriya.samarskaya2012@yandex.ru (V.O.S.); rysalka47@gmail.com (N.S.); szavriev@ibch.ru (S.K.Z.); nkalin46@mail.ru (N.O.K.); 2 Doka-Gene Technologies Ltd., 141880 Rogachevo, Russia; n.markin@dokagene.ru (N.M.); suprunova@gmail.com (T.P.S.); 3 The James Hutton Institute, Invergowrie, Dundee DD2 5DA, UK; andrew.love@hutton.ac.uk; 4 Belozersky Institute of Physico-Chemical Biology, Lomonosov Moscow State University, 119991 Moscow, Russia

**Keywords:** potato virus Y, RNA interference, pattern-triggered immunity, poly(ADP-ribose) metabolism

## Abstract

In this work we developed and exploited a spray-induced gene silencing (SIGS)-based approach to deliver double-stranded RNA (dsRNA), which was found to protect potato against potato virus Y (PVY) infection. Given that dsRNA can act as a defence-inducing signal that can trigger sequence-specific RNA interference (RNAi) and non-specific pattern-triggered immunity (PTI), we suspected that these two pathways may be invoked via exogeneous application of dsRNA, which may account for the alterations in PVY susceptibility in dsRNA-treated potato plants. Therefore, we tested the impact of exogenously applied PVY-derived dsRNA on both these layers of defence (RNAi and PTI) and explored its effect on accumulation of a homologous virus (PVY) and an unrelated virus (potato virus X, PVX). Here, we show that application of PVY dsRNA in potato plants induced accumulation of both small interfering RNAs (siRNAs), a hallmark of RNAi, and some PTI-related gene transcripts such as *WRKY29* (WRKY transcription factor 29; molecular marker of PTI), *RbohD* (respiratory burst oxidase homolog D), *EDS5* (enhanced disease susceptibility 5), *SERK3* (somatic embryogenesis receptor kinase 3) encoding brassinosteroid-insensitive 1-associated receptor kinase 1 (BAK1), and *PR-1b* (pathogenesis-related gene 1b). With respect to virus infections, PVY dsRNA suppressed only PVY replication but did not exhibit any effect on PVX infection in spite of the induction of PTI-like effects in the presence of PVX. Given that RNAi-mediated antiviral immunity acts as the major virus resistance mechanism in plants, it can be suggested that dsRNA-based PTI alone may not be strong enough to suppress virus infection. In addition to RNAi- and PTI-inducing activities, we also showed that PVY-specific dsRNA is able to upregulate production of a key enzyme involved in poly(ADP-ribose) metabolism, namely poly(ADP-ribose) glycohydrolase (PARG), which is regarded as a positive regulator of biotic stress responses. These findings offer insights for future development of innovative approaches which could integrate dsRNA-induced RNAi, PTI and modulation of poly(ADP-ribose) metabolism in a co-ordinated manner, to ensure a high level of crop protection.

## 1. Introduction

Given the versatile tunability of the properties and activities of RNA, novel RNA-based technologies are extensively emerging. A growing body of research confirm their successful applications via both host-induced (transgenic) gene silencing (HIGS) and spray-induced gene silencing (SIGS) techniques in plant protection against various plant pathogens, including viruses. Gene silencing or RNA interference (RNAi) is a pervasive nucleotide sequence-specific mechanism of gene regulation which involves production of small RNAs that induces the silencing machinery to target complementary DNA or RNA for transcriptional (TGS; i.e., nucleic acid methylation) or post-transcriptional (PTGS; i.e., degradation of specific RNAs for repression of virus genome translation) silencing, respectively [1,2,3,4]. In the context of RNA viruses, double-stranded RNA (dsRNA) molecules formed during virus replication are recognised and cleaved by Dicer-like proteins (DCL) into small interfering RNAs (siRNAs). Subsequently these siRNAs are loaded into a complex composed of ARGONAUTE (AGO) protein family members to form an activated RNA-induced silencing complex which acts to specifically degrade complementary viral RNAs [1,2,3,5,6,7]. Host RNA-dependent RNA polymerases (RDRs) such as RDR6 also play an important role in antiviral RNA silencing by amplifying the synthesis of virus-derived dsRNA molecules [8]. RNAi-mediated antiviral immunity acts as a major virus resistance mechanism in plants.

The commercial development of transgenic crops with engineered disease resistance is constrained by regulations around the genetic modification (GM) of plants, environmental concerns and negative public perception. Thus, there is a demand for alternative approaches for crop protection which are more sustainable, effective, environmentally friendly and positively perceived. SIGS technology based on the spray application of exogenous dsRNA to plants [9,10,11] could offer this, and moreover, this approach has already been successfully deployed to induce resistance to different viruses in a wide range of crops [9,10,11]. The key role of RNAi as an underlying mechanism in exogeneous dsRNA-mediated antiviral protection has been confirmed by reports showing that the exogenously delivered dsRNA interferes with virus infection in a sequence-specific manner: for example, potato virus Y (PVY)-specific dsRNA protected tomato plants from PVY but not from tomato mosaic virus (ToMV) and vice versa [12]; in addition, dsRNA complementary to the gene for green fluorescent protein (GFP) did not provide resistance to tomato spotted wilt virus (TSWV; [13]). Interestingly, exogenously applied long dsRNA remained in the treated leaves for at least 10 days, but its systemic movement was not observed. However, dsRNA-derived siRNA populations (mainly consisting of 21 nt-long and 22 nt-long siRNAs) were detected in non-treated leaves, which clearly demonstrates their endogenous processing and systemic spread through the plant. In another report, Necira et al. [14] showed that *Nicotiana benthamiana* plants could also be protected from PVY and potato virus X (PVX) by using corresponding exogenous dsRNA targeting the homologous virus (either PVY or PVX). Moreover, it was previously indicated that host-encoded RDR6 and the combined activities of DCL2 and DCL4 are required to promote efficient resistance to virus infection conferred by topical application of dsRNA [14]. DCL4 generates 21 nt siRNAs and is the primary DCL component of antiviral defence against RNA viruses, while DCL2 generates 22 nt secondary siRNAs which trigger transitivity and, as such, expands the silencing signal to additional sequences [14]. As mentioned above, RDR6 also plays an essential role in antiviral RNA responses by amplifying the synthesis of dsRNA molecules in plants [8,14]. These data demonstrate that exogenous dsRNA molecules are processed and exploited by the RNAi pathways commonly used by plant hosts in response to virus infections.

In addition to its role in inducing RNAi, dsRNA has been reported to be an elicitor of pattern triggered immunity (PTI; [15,16,17]). RNA-based pathogen-associated molecular patterns (PAMPs) are well-known inducers of immunity in both animals [18,19,20,21], and plants [15,22]. The defence responses invoked by viruses and dsRNA are canonical to those of antimicrobial PTI [23], and include the production of reactive oxygen species (ROS), induction of hormone signalling, the activation of mitogen-activated protein kinases, and the triggering of defence gene expression [15,24,25,26]. Unlike RNAi, dsRNA-induced PTI is RNA sequence independent and may also be activated by non-viral dsRNA, such as synthetic polyinosinic:polycytidilic acid (poly (I:C)) or GFP-specific dsRNA [15]. For example, poly (I:C) which induced PTI markers also elicited strong antiviral defence against oilseed rape mosaic virus (ORMV; [15]), which was not contingent on nucleic acid sequence similarity to the virus. The mechanisms of dsRNA-mediated PTI action against infecting viruses remains largely uncharacterised. A possibility is that dsRNA inhibits the progression of virus infection via PTI-directed callose deposition at intercellular cytoplasmic channels (plasmodesmata), which could concomitantly inhibit cell-to-cell movement of the viral components through these plant-specific communication structures [17]. Interestingly, dsRNA-induced host responses such as callose deposition at the plasmodesmata can be suppressed by viral movement proteins (MPs) [17].

Collectively, all these observations suggest that hypothetically two mechanisms induced by dsRNA, namely, sequence-specific RNAi and sequence-independent PTI, may contribute to plant protection against viruses during SIGS. However, while the role of RNAi as a major antiviral mechanism during exogeneous dsRNA applications is thoroughly investigated and proved [9,10,11,27,28], the molecular inputs and outputs of dsRNA-mediated PTI in plant antiviral protection is only just beginning to be unravelled. To obtain further insights into the mechanisms underlying antiviral action of exogenous dsRNA, we explored the activities of PVY-specific dsRNA in inducing resistance to homologous (PVY) and unrelated (potato virus X, PVX) viruses and investigated the concomitant activation of various defence mechanisms including RNAi and PTI.

PVY RNA was chosen for this study because PVY (the type member of the genus *Potyvirus*) is one of the most economically important pathogens of potato [29]. It significantly affects potato yield and quality: in the case of secondary infections, for example, yield reductions can reach up to 85% [30]. One of the important means to control PVY is the deployment of resistant cultivars [29]. However, conventional breeding to incorporate major resistance genes even with new genetic marker technologies is a time-consuming and laborious process. Novel emerging technologies such as SIGS represents an opportunity to trigger the natural plant virus (PVY) control system by providing rationally designed external dsRNA, which can be easily/rapidly deployed for crop protection.

The main goal of this study is to explore the molecular mechanisms underlying antiviral activity mediated by externally delivered virus-specific dsRNA in plants. In this work, we developed and exploited a SIGS-based approach in the application of dsRNA to protect potato from PVY. Given that dsRNA can act as a defence-inducing signal for both RNAi and PTI, we aimed at testing the impact of exogenous applied PVY-derived dsRNA on both these layers of defence. We have shown that hallmarks of both RNAi (such as siRNA; [5]) and PTI, the latter of which is typified by increased PTI-related genes such as *WRKY29* (WRKY transcription factor 29, a molecular marker of PTI; [31]), *PROPEP3* (elicitor peptide 3 precursor); [15]), *EDS5* (enhanced disease susceptibility 5; [15]) and *PR-1b* (pathogenesis-related gene 1b; [15,32]), are induced by PVY dsRNA in potato. However, PVY dsRNA suppressed only PVY replication but did not exhibit any effect on PVX infection in spite of PTI-like effects induced in the presence of PVX. Given that RNAi-mediated antiviral immunity acts as a major virus resistance mechanism in plants, it can be suggested that dsRNA-based PTI alone may not be strong enough to suppress virus infection. In addition to RNAi- and PTI-inducing activities, we have also found that PVY-specific dsRNA, like some other canonical elicitors of PTI, such as the peptide fragment of bacterial flagellin (flg22) or translation elongation factor Tu (elf18), is able to modulate poly(ADP-ribose) metabolism [33]. PolyADP-ribosylation (PARylation) is a post-translational modification of proteins in which chains of ADP-ribose are added to a target protein by enzymes known as poly-ADP-ribose polymerases (PARPs). PARylation plays important roles in genotoxic stress tolerance and DNA repair, transcription, cell cycle control and cellular responses to biotic and abiotic stresses including programmed cell death-related processes and regulation of plant immunity [34,35,36,37,38,39,40]. Poly-ADP-ribose (PAR) moieties may be removed/degraded by poly-ADP-ribose glycohydralase (PARG) enzymes, which are regarded as positive regulators of biotic stress tolerance. Interestingly, in our study, we found that PVY dsRNA upregulated PARG production in potato plants, which correlated with alterations in PAR accumulation. Taken together, these findings offer insights for future development of innovative approaches which could integrate dsRNA-induced RNAi, PTI and modulation of poly(ADP-ribose) metabolism in a co-ordinated manner, to ensure high level of crop protection.

## 2. Results

### 2.1. Specific Protection against PVY Infection by External dsRNA Application

**Figure 1:**
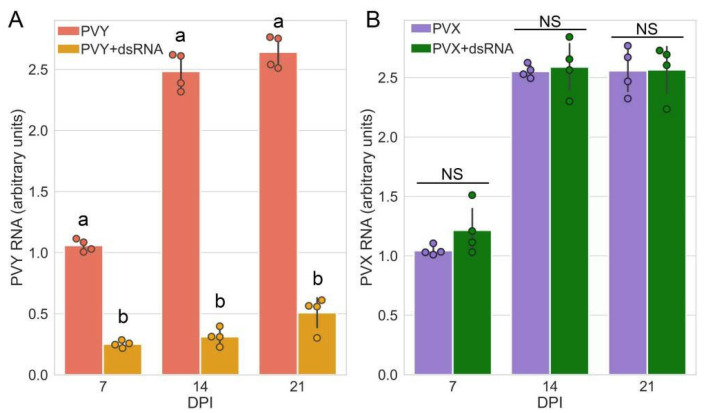
Accumulation of PVY RNA (**A**) and PVX RNA (**B**) (measured using RT-qPCR) in systemically infected leaves of potato plants cv. Indigo untreated or pre-treated with PVY dsRNA over a 7–21 dpi time periods as shown. PVY RNA and PVX RNA expression levels were normalized to those of internal controls, *StEF-1α* and *StCox.* Statistical analysis was performed on four independent biological replicates. Data are mean ± SD. Each replicate was composed of samples from three plants pulled pooled together (two leaves per plant). ANOVA and Tukey’s HSD post hoc tests were performed for RT-qPCR data. The different letters (a, b) indicate significantly different values (*p* < 0.001) in PVY RNA accumulation between dsRNA-treated and -untreated plants in a given time point. NS, not significant.

The ability of dsRNA molecules to induce protection against PVY (strain O) was tested in *Solanum tuberosum* (potato) plants cv. Indigo using bacterially produced dsRNA molecules homologous to the conservative fragment of the PVY replicase gene [41], which has a length of 918 bp (nts 7338–8255; Gene Bank accession number AB711155.1). PVY-dsRNA was applied to the plants in the presence of an abrasive (celite) and surfactant (Neon 99) using an atomizer sprayer. PVY was inoculated 24 h later. Treatment of the plants with dsRNA was repeated weekly. Application of dsRNA resulted in efficient inhibition of viral propagation in systemically infected leaves of the treated plants until at least 21 days post-inoculation (dpi) compared with the control (plants buffer-treated rather than sprayed with dsRNA) (Figure 1A).

**Figure 2:**
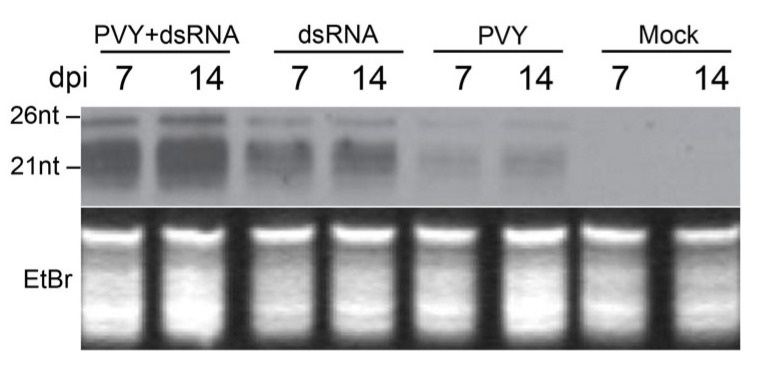
Production of PVY-specific siRNAs induced by exogenous PVY dsRNA application in mock or systemically infected PVY plants (7 and 14 days post treatment as shown). Mock or PVY infected plants not treated with dsRNAs were used as controls. Ethidium bromide (EtBr) stained 5S rRNA+tRNA samples are shown as loading controls. Positions of 26 and 21 nt-size markers are indicated. Uncropped image is presented in Supplementary Figure S1. Similar results were obtained using four independent samples described in Figure 1 experiment.

The numerous reports describing anti-viral defences induced by external dsRNA application (SIGS) have suggested that the protective effect of exogenous of dsRNAs is based on the RNAi-mediated response, interfering with virus infection in a sequence-specific manner (for reviews, see [9,11]). Analysis of the low-molecular-weight RNA in samples via Northern blot analysis using probes derived from PVY RNAs confirmed that PVY-dsRNA itself was able to induce high levels of PVY-specific siRNAs (which are the main hallmark of RNAi) which even exceeded those in PVY-infected plants in the absence of dsRNA (Figure 2). In the presence of dsRNA, PVY infection resulted in over-accumulation of siRNAs, which strongly correlated with a remarkable reduction in PVY viral RNA (Figure 1A), supporting the assumption that RNAi is involved in dsRNA-mediated protection against PVY.

In the next series of experiments, we evaluated the specificity of PVY-dsRNA protection. In contrast to PVY infection (Figure 1A), when potato plants were inoculated with PVX post PVY-dsRNA treatment, no protection was observed (Figure 1B). These results are consistent with the sequence-specific manner of RNAi-based antiviral protection.

### 2.2. PVY-Specific dsRNA Induces Typical PTI Responses in Potato

**Figure 3:**
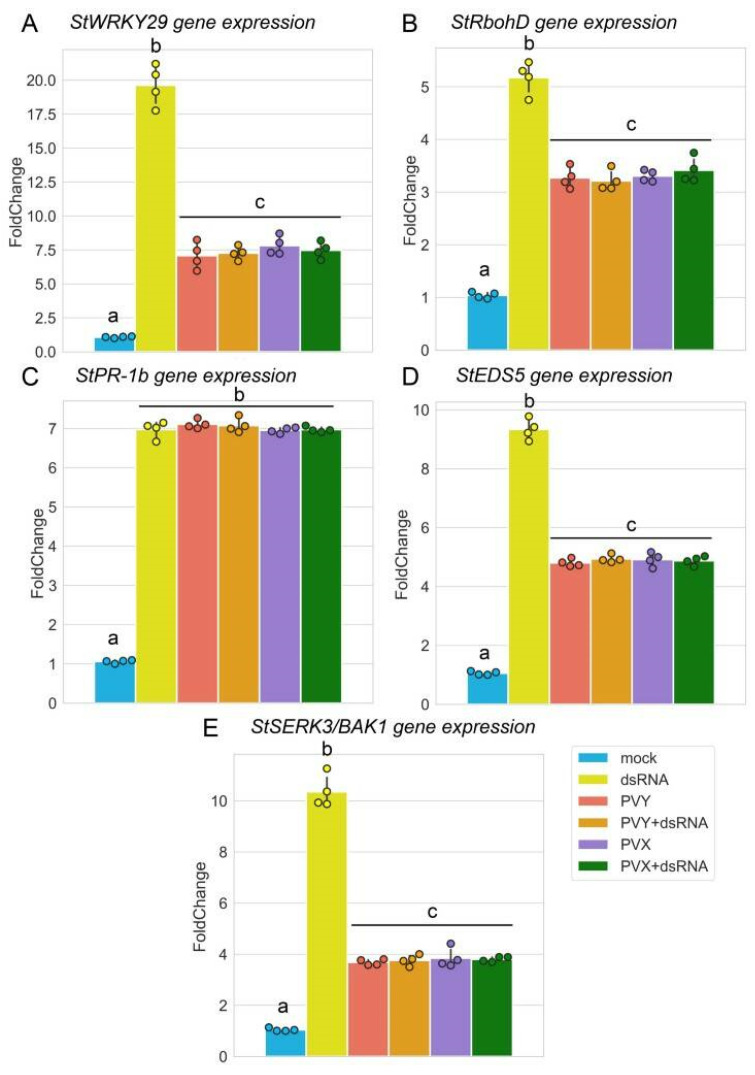
Modulation of PTI-based defence responses in potato plants cv. Indigo to PVY or PVX infection via application of PVY dsRNA. (**A**–**E**) Expression patterns of PTI-related genes, *StWRKY29* (**A**), *StRbohD* (**B**), *StPR-1b* (**C**), *StEDS5* (**D**), and *StSERK3/BAK1* (**E**) analysed by RT-qPCR in systemically or mock infected leaves at 7 days post treatment. *StWRKY29*, WRKY transcription factor 29; *StRbohD*, respiratory burst oxidase homolog D; *StPR-1b*, pathogenesis-related gene 1b; *StEDS5*, enhanced disease susceptibility 5; *SERK3/BAK1*, somatic embryogenesis receptor kinase 3 encoding brassinosteroid-insensitive 1-associated receptor kinase 1; *PR-1b*, pathogenesis-related gene 1b. mRNA expression levels were normalized to those of internal controls, *StEF-1α* and *StCox*. Statistical analysis was performed on four independent biological replicates. Data are mean ± SD. Each replicate was composed of samples from three plants pulled pooled together (two leaves per plant). Analysis of variance and Tukey’s HSD post hoc tests were performed on the data obtained. The different letters (a, b, c) indicate significantly different values (*p* < 0.001).

**Figure 4:**
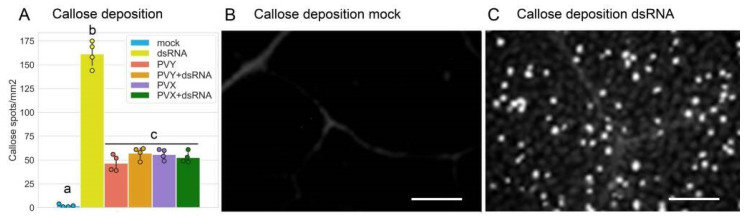
Effect of PVY dsRNA on callose deposition in potato plants cv. Indigo infected with PVY or PVX. (**A**) Quantitative evaluation of callose deposition in systemically or mock infected leaves at 7 days post treatment. Statistical analysis was performed on four independent biological replicates. Data are mean ±SD. Analysis of variance and Tukey’s HSD post hoc tests were performed on the data obtained. The different letters (a, b, c) indicate significantly different values (*p* < 0.001). (**B**,**C**) Images of callose deposition (visualized by aniline blue staining) in potato leaves at 7 days post treatment exemplified by a leaf tissue of mock-infected plants non-treated ((**B**), served as a control) and treated (**C**) with dsRNA. Scale bar, 50 μm.

It has been demonstrated that dsRNAs of various origins, such as GFP-specific dsRNA, dsRNA purified from OSMV-infected plants or synthetic analogues of dsRNA (poly (I:C)), all are able to induce typical PTI responses in Arabidopsis [15]. To test virus-specific dsRNA as an elicitor of PTI in potato, we examined whether PVY-specific dsRNA can induce PTI-like signalling pathways in this crop plant. Potato PTI-responsive candidate genes were selected based on a bibliographical search of those genes induced by canonical PTI elicitors such as flg22, elf18 (translation elongation factor Tu) or Pep25 (an oligopeptide fragment of a *Phytophthora* sojae 42-kDa cell wall protein). We found that PVY dsRNA could trigger expression of a range of classical PTI-related genes such as *WRKY29* (WRKY transcription factor 29; a molecular marker of PTI; [42]), *RbohD* (respiratory burst oxidase homolog D; [43], *EDS5* (enhanced disease susceptibility 5; [44]), *SERK3* (somatic embryogenesis receptor kinase 3) encoding brassinosteroid-insensitive 1-associated receptor kinase 1 (BAK1) [45]), and *PR-1b* (pathogenesis-related gene 1b; [42]) (Figure 3A–E). Given that mechanisms underlying dsRNA-mediated PTI in Arabidopsis and *Nicotiana benthamiana* triggers callose deposition at plasmodesmata which may inhibit virus cell-to-cell movement [17], we examined whether PVY-dsRNA may also increase callose levels in potato. Figure 4A–C shows that potato also responds to treatment with PVY-dsRNA with enhanced callose deposition.

Interestingly, PVY and PVX themselves also facilitated expression of the same PTI-responsive genes (as mentioned above; Figure 3A–E) as well as callose deposition (Figure 4), although to lower levels than PVY-dsRNA alone, suggesting that PTI signalling pathways may be a genuine plant response to these viruses. At the same time both PVY and PVX significantly decreased the capacity of dsRNA to induce callose deposition and expression of all the tested PTI-responsive genes, apart from *PR-1b* (Figure 3 and Figure 4). This is consistent with the idea that plant viruses have evolved virulence strategies to suppress PTI-based host defences [46]. Indeed, some virus-encoded proteins such as the cauliflower mosaic virus (CaMV) P6 [25,47] or movement protein (MP) of tobacco mosaic virus (TMV) can act as PTI suppressors.

Taken together, our findings suggest that in addition to RNAi, PVY-dsRNA induces PTI signalling pathways. However, while the RNAi-based defence mechanisms in this case operate efficiently by inhibiting PVY infection in a sequence-specific manner, PTI responses on their own may not be strong enough to protect plants from unrelated viruses (PVX).

### 2.3. Effect of PVY-Specific dsRNA on Poly(ADP-Ribose) Metabolism

As discussed earlier, polyADP-ribosylation (PARylation) of proteins by PARP and removal of these moieties from the proteins by PARG, is an important switch influencing plant immunity [34,35,36,37,38,39,40], with PARG being regarded as a positive regulator of biotic stress tolerance.

Arabidopsis encodes two PARG genes, PARG1 and PARG2 [35]. Interestingly, AtPARG1, but not AtPARG2, carries glycohydrolase activity in vivo and in vitro. Treatment of Arabidopsis plants with the classical PAMP, flg22, transiently induced PARG1 mRNA and upregulated expression of PARG2 mRNA [48,49]. PARG2 transcripts were also upregulated in cucumber mosaic virus (CMV)-resistant plants [50]. However, genetic abolishment of PARG1 in knockout Arabidopsis parg1 plants enhanced flg22-induced callose deposition and increased expression of flg22-regulated genes [38]. At the same time, the onset of symptoms caused by Botrytis cinerea was accelerated in both parg1 and parg2 mutant plants [49]. Taken together, these observations establish an intriguing link between poly(ADP-ribose) metabolism and PTI which may not be assigned to a specific pathogen-induced signalling pathway, rather suggesting that PARGs can operate as a regulatory element of integrated defence network.

To examine whether dsRNA, like flg22, can induce changes in the expression of PARylation-associated genes, the transcriptional expressions of *PARP* and *PARG* genes were analysed by RT-qPCR in potato samples. As a PARP family member, we selected *StPARP1* (Soltu.DM.03G032200.1) homologous to *AtPARP1* which acts as the predominant PARP in Arabidopsis [51]. As a PARG family member, *StPARG* (Soltu.DM.12G003820.1) was chosen as it possessed a signature motif with the conserved sequence of ‘‘GGG-X7-QEE’’ which is required for PAR glycohydrolase activity is presented in AtPARG1, but not in AtPARP2 [38].

**Figure 5:**
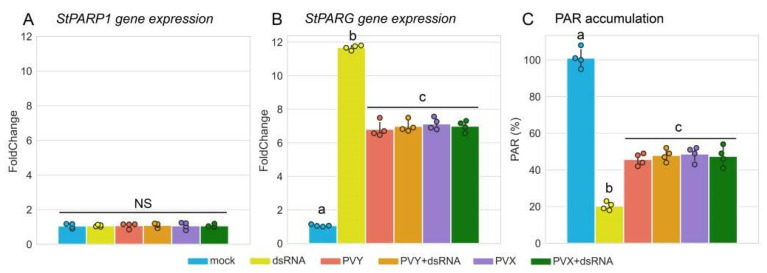
Modulation of poly(ADP-ribose) metabolism in potato plants cv. Indigo by PVY dsRNA in the presence or absence of PVY or PVX infection. (**A**,**B**) The expression patterns of genes encoding poly(ADP-ribose) polymerase (StPARP1, (**A**)) and poly(ADP-ribose) glycohydrolase (StPARG, (**B**)), analysed by RT-qPCR in systemically or mock infected leaves of Indigo plants at 7 days post treatment, as shown. mRNA expression levels were normalized to those of internal controls, *StEF-1α* and *StCox.* Statistical analysis was performed on four independent biological replicates. Data are mean ±SD. Each replicate was composed of samples from three plants pulled pooled together (two leaves per plant). (**C**) Accumulation of PARylated proteins measured by ELISA using rabbit anti-PAR polyclonal antibody, in systemically infected or mock-inoculated plants at 7 days post treatment. Statistical analysis was performed on four independent biological replicates. Analysis of variance and Tukey’s HSD post hoc tests were performed on the data. The different letters (a, b, c) indicate significantly different values (*p* < 0.001).

Neither PVY-dsRNA itself, nor PVY or PVX in the presence or absence of dsRNA affected expression of *PARP1* (Figure 5A). In contrast, *PARG* expression was significantly upregulated by treatment with PVY-dsRNA compared with untreated plants (Figure 5B). PVY and PVX themselves also facilitated expression of *PARG* although to a lesser extent than PVY-dsRNA alone. Nevertheless, both PVY and PVX applied together with PVY-dsRNA decreased the capacity of dsRNA to induce *PARG* and had similar levels of induction to that of the viruses alone (Figure 5B).

Taken together these results indicate that *PARG* overexpression in PVY dsRNA-treated potato plants correlates well with increased callose deposition and expression of PTI-related genes (Figure 3 and Figure 4). Notably, as mentioned above, the same effects were observed in *PARG1* knockout (*parp1*) Arabidopsis lines treated with flg22 [38]. This could indicate that the particular dsRNA/virus treatments exploited in this work may uncouple the functioning of the PARG-callose signal transduction observed with flg22-treated *parg1* Arabidopsis mutants.

PARG is the primary hydrolase involved in the degradation of PAR and is believed to play an important role in preventing excessive accumulation of PAR derived from proteins PARylated by PARP (target proteins). Therefore, it could be expected that PAR levels might be significantly decreased following upregulation of PARG expression. Indeed, potato plants treated with PVY-dsRNA in the presence or absence of PVY or PVX had high levels *PARG* expression and a consistent low level of PAR, which is suggestive of the negative correlation between the two (Figure 5C). It is thus intriguing to speculate on whether exogenous dsRNA-mediated *PARG* transcriptional activation in potato and subsequently reduced PARylation may at least partially regulate PTI responses.

## 3. Discussion

The antiviral effect of exogenous dsRNA is usually attributed to RNAi silencing-mediated plant defence [9,10,11]. However, it has been suggested that dsRNA may also be involved as an elicitor in PTI, another host defence mechanism operating in virus-infected plants [15,16,17]. While the role of RNAi as a major defence mechanism during exogeneous dsRNA applications is thoroughly elucidated (for reviews, see [9,10,11,27,28]), the molecular mechanisms underlying dsRNA-mediated PTI in plant antiviral protection still remain elusive.

First of all, it is unclear how dsRNA may be perceived during PTI. Unfortunately, parallels between classical (antimicrobial) and antiviral PTI may only partially provide insights into mechanisms of dsRNA recognition and perception during immune responses [16]. PTI against microbial pathogens typically involves plasma membrane-bound receptors. It is possible that when the dsRNA is delivered via external application, it enters into the apoplast and may then be specifically perceived by a plasma membrane-associated PAMP recognition receptor. However, it is also reasonable to assume that this dsRNA will also be found intracellularly, given that this localization is required for its expected RNAi activity. It is also an obvious assumption that dsRNAs produced during natural virus infections will be found in an intracellular location. It is possible that PTI may also be mediated via intracellular dsRNAs which could associate with putative membrane-localized dsRNA PAMP receptors which can presumably cycle between the plasma membrane and endosomal compartments [16]. Finally, it cannot be excluded that PTI receptors for dsRNA PAMPs may be located in the cytoplasm [16].

Regardless of the perception mechanism, dsRNA is recognised as a PAMP in potato and induces typical PTI responses. Among the classical PTI-related genes upregulated during dsRNA application in potato are: WRKY29, which encodes a transcription factor involved in the expression of defence genes in plant innate immune responses [42]; RbohD, which encodes a respiratory burst oxidase homolog D which acts as a key driver of reactive oxygen species signalling by integrating various signal transduction pathways in plants [43]; EDS5, which encodes an essential component of salicylic acid-dependent defence signalling for disease resistance [44]; SERK3, which encodes a brassinosteroid-insensitive 1-associated receptor kinase 1, which is considered to be a node which integrates different signalling pathways, due to its important role in the perception of exogenous and endogenous cues [45]; PR-1b, which encodes a PR-protein which is a traditional hallmark of activation of the SA-mediated signalling pathway in potato [42]. Interestingly, in an earlier report [15], the authors did not observe any effect of dsRNA on expression of the PR-1 gene. This discrepancy may be attributed to the differences in the experimental methods, plant species and time points employed in these studies.

It should be noted that the dsRNA-induced signalling pathways lead to callose deposition presumably at plasmodesmata, intercellular channels used by viruses for cell-to-cell movement. However, the question is bound to arise as to why exogenous dsRNAs, which appear to induce PTI in a non-sequence-specific manner, do not confer noticeable antiviral protection against unrelated viruses (i.e., [12,13]; Figure 1 in this work).

DsRNA serves as a trigger for both PTI and RNAi, pathways that may be mutually exclusive, although the exact determinants responsible for the shift from RNAi to PTI have not yet been identified. The simplest assumption for the lack of dsRNA mediated protection against unrelated viruses (PTI) may be potential rivalry between PTI and RNAi pathways. It is quite likely that highly efficient dsRNA-mediated RNAi processes can merely outcompete PTI for dsRNA populations, re-attracting most of dsRNA pool from PTI receptors to its own machinery. In such a case, exogenously applied dsRNA (in our case PVY-dsRNA) can mainly be absorbed by the RNAi machinery, inducing RNAi-based defence against homologous viruses (PVY). This will inevitably diminish (or even exclude) interaction of dsRNA with the PTI signalling pathway and, as a result, prevents PTI-based protection even against unrelated virus (PVX).

Interestingly, viruses, regardless of their homology to dsRNA, may further enhance dsRNA-mediated RNAi efficiency by upregulating critical RNAi components such as DCL, AGO and RDR [52], which in turn may prevent (or reduce) utilization of dsRNA as an elicitor of PTI. It can be speculated that homopolymeric double-stranded polyribonucleotide poly (I:C) may not be recognized and cleaved by DCL proteins and hence may be excluded from the RNAi pathway. Thus, in contrast to natural dsRNAs, which are strongly dedicated to RNAi, poly (I:C) may be fully involved in the PTI pathway, conferring strong antiviral resistance as shown by [15]. Thus, balanced partitioning of the dsRNA pool to the interdependent pathways competing for dsRNA (RNAi and PTI) seems to be required to ensure robust and efficient antiviral protection.

An additional possibility may be attributed to putative negative regulatory effects of RNAi on the PTI machinery. Indeed, numerous nucleotide-binding and leucine-rich repeat (NB-LRR) resistance genes involved in PTI are post-transcriptionally regulated by conversion of their transcripts into dsRNA by RDR6 and subsequent cleavage into siRNAs by DCL proteins [53,54,55]. Thus, the induction of antiviral RNAi could significantly facilitate the concomitant down-regulation of PTI genes.

Another intriguing finding presented in this paper is a previously unrecognized activity of exogenously applied dsRNA to modulate poly(ADP-ribose) metabolism. In plants, PARylation events have been implicated in various biological processes such as DNA damage responses, maintenance of genome integrity, chromatin remodelling, cell death, development, metabolism and responses to biotic and abiotic stresses [33]. Genetic studies in Arabidopsis showed that inhibition of the PARG1 gene may significantly modify expression of PAMP-induced (PTI-related) genes [40]. Such responses may indicate functional roles for PARG and PARylation in innate plant immunity (PTI). However, the molecular mechanisms underlying these effects remain largely unknown. Here, we demonstrated that PARG gene expression in potato is strongly upregulated by exogenous PVY-dsRNA application either in the absence or in the presence of homologous (PVY) or unrelated (PVX) virus in potato, and this leads to decreased rates of PARylation. Infection with PVY or PVX themselves upregulated PARG expression, but to a lesser extent than dsRNA alone.

Although there is compelling evidence that dsRNA can modulate poly(ADP-ribose) metabolism, future research is required to elucidate the role of PARG and PAR metabolism in the context of processes induced by exogenous dsRNA applications. It would be important, for example, to identify PARylation acceptors that are specifically targeted during dsRNA applications and their possible role in plant defence. It is also important to develop approaches to fine-tune dsRNA-mediated PARylation as a crucial process required for various biological functions, but excessive PARylation is highly toxic to the plants [35,40].

In summary, exogenous dsRNA application is a multi-faceted technology which mainly triggers RNAi, the major virus resistance pathway, and also induces PTI/PAR-based mechanisms which may represent a safeguard strategy in case the RNAi mechanisms fail to be effective to counter virus infection (although the PTI/PAR mechanisms may still fail if a particular virus encodes effectors which can switch off PTI/PAR responses). It can also be speculated that PTI/PAR-based pathways may prevent colonisation by ancillary opportunistic pathogens, or modulate susceptibility to different stresses in order to promote plant survival. Therefore, innovative biotechnology approaches which could integrate PTI/PAR-based pathways and RNAi in a co-ordinated manner could ensure a high level of sustainable crop protection from viruses.

## 4. Materials and Methods

### 4.1. Virus, Plants, and Growth Conditions

PVY (ordinary strain, PVY-O) and PVX (Russian strain, PVX-RU) were propagated in *Nicotiana tabacum*. Four-week-old potato plants (*Solanum tuberosum* L.; cv. Indigo) were inoculated with PVY or PVX and left to grow in a controlled environment chamber (Pol-Eko-Aparatura, Wodzisław Śląski, Poland) which was set at a photoperiod of 16/8 h day/night at a relative humidity of 60% with a light fluence of 250 μmol m^−2^·s^−1^.

### 4.2. Production and Purification of dsRNA

cDNA corresponding to the conservative fragment of the PVY replicase gene [41], which has a length of 918 bp (nts 7338–8255; Gene Bank accession number AB711155.1), was synthesized by Evrogen (Moscow, Russia) and cloned into the plasmid vector L4440 (plasmid Addgene 1654). This vector contains two T7 promoters in an inverted orientation that flanks the multiple cloning sites. The recombinant L4440 vector was transformed into HT115 (DE3) *Escherichia coli* HT115 strain via standard transformation procedures [56]. This strain does not produce RNase III, a dsRNA degradation enzyme. T7 RNA polymerase-mediated transcription was induced with isopropyl β-d-1-thiogalactopyranoside (IPTG). The bacterial cultures were centrifuged at 5000× *g* for 10 min at 4 °C. After discarding the supernatant, the bacterial pellet was resuspended in NH_4_OAc (1 M) with 10 mM EDTA and dsRNA was extracted using conventional phenol/chloroform approaches [56]. To increase the yield of accurately assembled RNA duplexes, the generated dsRNA in 10 mM Tris-HCl pH 7.5, 2.5 mM MgCl_2_, 0.1 mM CaCl_2_ was heated for 5–10 min at 95 °C and gradually cooled down to room temperature (over a period of no less than 30 min). The purified dsRNA was quantified using a NanoDrop 2000 spectrophotometer and the integrity of the dsRNA was evaluated by electrophoresis through a 1.2% TBE-agarose gel.

### 4.3. Exogenous dsRNA Application for Plant Protection against Virus Infection

The PVY-dsRNA solution in distilled water (45 μg per plant) was applied to the plants in the presence of 2000-fold diluted surfactant (Neon 99) using an atomizer sprayer. Then, 24 h after dsRNA or buffer application (including surfactant), the plants were challenged by mechanical inoculation of the viruses. Plants inoculated with the virus without the dsRNA application, and mock-inoculated plants were used as controls, respectively. Each test consisted of 6 plants per treatment, replicated four times.

### 4.4. Plant RNA Extraction and Northern Blot Analysis

Leaf tissues (1 to 2 g) were frozen in liquid nitrogen and ground to fine powder in a mortar and pestle, and total RNA was extracted using TRI REAGENT according to the manufacturer’s recommendations (Sigma-Aldrich, St. Louis, MO, USA). RNA was suspended in 50 μL DEPC-treated water. Low-molecular weight (LMW) RNAs were isolated and resolved by electrophoresis in polyacrylamide/7M urea gels, which were then electroblotted onto Hybond N membranes prior to UV cross-linkage using a StrataLinker (Stratagene, La Jolla, CA, USA) as described by Taliansky et al. [57]. The [^32^P]-labelled RNA probes corresponding to the 918 bp (nts 7338–8255) fragment of PVY RNA were generated from PCR products obtained from the recombinant L4440 vector described above, using the mMESSAGE mMACHINE T7 kit (Ambion Inc., Austin, TX, USA) with a random-priming DNA labelling system (Invitrogen). As a loading control, equal fractions of each sample were resolved on a 1% agarose gel and stained with ethidium bromide (EtBr). Similar results were obtained in three independent experiments.

### 4.5. Real Time Quantitative RT-PCR (RT-qPCR)

Species-specific prefixes (St) are used in the text and Supplementary Table S1 to define the *S. tuberosum* genes: *StWRKY29*, *StRbohD*, *StPR-1b*, *StEDS5*, *StSERK3/BAK1*, *StPARP1*, *StPARG*, *StEF-1α* and *StCox*. However, for simplicity, in places in the manuscript this “St” nomenclature is not used for genes, proteins or mRNAs. Total RNA was isolated as described above. DNase-treated RNA was reverse-transcribed into cDNA using the SuperScriptTM First-Strand Synthesis System for RT-PCR (Invitrogen), with either an oligo-dT primer (for host plant-specific mRNAs) or PVY- or PVX-specific primers (see Table S1). The primer pairs for SYBR green-based real-time PCR analysis of PVY RNA, PVX RNA and host mRNAs (which are listed in Table S1) were designed using both Plant Genomics Resource Phytozome (https://phytozome.jgi.doe.gov/pz/portal, accessed on 25 May 2022) and PRIMER EXPRESS software (ThermoFisher Scientific, Waltham, MA, USA). The Ct values for PVY RNA, PVX RNA and each mRNA of interest were normalized using two internal reference genes encoding StEF-1α [58] and cytochrome c oxidase subunit 1 (StCOX) [59]. The average Ct values of the two reference genes were used to analyse PVY and host mRNA levels.

### 4.6. Callose Staining

To detect callose, potato leaves were cleared with 95% ethanol overnight and stained with 150 mM K_2_P0_4_ (pH 9.5), 0.01% aniline blue for 2 h [60]. Leaves were examined with a Leica MZ FL III Fluorescence microscope. The number of callose deposits were counted using ImageJ 1.43U software (http://rsb.info.nih.gov/ij/, accessed on 25 May 2022). More than 10 adjacent fields of view along the length of the leaf (not including the midvein or leaf edge) were analysed and averaged.

### 4.7. Immunological Detection of Poly(ADP-Ribose) (PAR)

For the isolation of plant nuclei and nuclear protein extraction the CelLytic PN kit (Sigma-Aldrich, St. Louis, MO, USA) was used [61]. The protein (5 µg) was analysed for PAR accumulation levels by ELISA using a purified monoclonal antibody (Trevigen, Gaithersburg, MD, USA) to PAR as the capture reagent, a rabbit anti-PAR antibody (Trevigen, Gaithersburg, MD, USA) as the detecting agent, and a goat anti-rabbit antibody conjugated with alkaline phosphatase (Sigma-Aldrich, St. Louis, MO, USA) as the reporter [62]. PAR polymer (Trevigen, Gaithersburg, MD, USA) was used as a positive control.

### 4.8. Statistics

Statistical analysis was performed on four independent biological replicates. Each replicate was composed of samples from three plants pulled pooled together (two leaves per plant). Statistical analyses and bar plots were made using Python version 3.7.5 [63]. For two- or multiple-way ANOVA, Tukey honestly significant difference (HSD) tests based on multiple comparisons of means were deployed to determine the pairwise comparisons which were statistically significant. Differences were considered to be significant if the *p*-value was <0.05.

## Data Availability

Not applicable.

## Supplementary Materials

The following supporting information can be downloaded at: https://www.mdpi.com/article/10.3390/ijms23147915/s1. References [64,65] are cited in the Supplementary materials.
