# Supplementary material for: Impact of Exogenous Application of Potato Virus Y-Specific dsRNA on RNA Interference, Pattern-Triggered Immunity and Poly(ADP-ribose) Metabolism
Source: Int J Mol Sci. 2022 Jul 18;23(14):7915. doi: 10.3390/ijms23147915 (PMC9317112; doi:10.3390/ijms23147915)
Supplement: Supplementary file 1 [file ijms-23-07915-s001.zip › ijms-1801170-supplementary.pdf]

**Table S1.** Primers used for quantitative RT PCR

| Primer | 5'-3' sequence | Reference | Primer concentration (nM) | E (%) |
|---|---|---|---|---|
| PVY$^{O}$-F<br>PVY$^{O}$-R | TATGATGGATTTGGCGACCACTTGT<br>TAAACTAGGCAGCTCTGCATCATG | Makarova et al., 2018 [32] | 400 | 95.7 |
| PVX-F<br>PVX-R | AACTGGCAAGCACAAGGTTTCA<br>CAGTTTGGGCAGCATTCATTTC | Du et al., 2006 [64] | 350 | 97.5 |
| StWRKY29 -F<br>StWRKY29-R | TGAACAAAAGAGAGTGGTGCTTCA<br>TTTTCTCCAAGCCCATTTATCAG | Lacaze and Joly, 2022 [42] | 400 | 98 |
| StPR-1b-F<br>StPR-1b-R | GTATGAATAATTCCACGTACCATATGTTC<br>GTGGAAACAAGAAGATGCAATACTTAGT | Baebler et al., 2011[59] | 350 | 100 |
| StEDS5-F<br>StEDS5-R | GGACCTTTGATGAGTCTTATTG<br>CATGCCAAGCCTCGAATCTG | Pajerowska et al., 2005 [44] | 400 | 96.5 |
| StRbohD-F<br>StRbohD-R | CGGTTTCAATGCCTTTTGGT<br>TGGACTATGAGGAGAGAATAGACAATG | Lacaze and Joly, 2022 [42] | 400 | 98 |
| StSERK3-F<br>StSERK3-R | TGTTTGGCTACGGAGTTATGC,<br>GCAAGTCGAGCAAGATCAAA | Nietzschmann et al., 2019 [45] | 400 | 95.5 |
| StPARP1-F<br>StPARP1-R | GCCATGGAAAGCTGAGTATG<br>GCCACATCGGCATTAATCCATC | Glushkevich et al., 2022 [65] | 400 | 95.4 |
| StPARG-F<br>StPARG-R | AGGAAGAAATTCGATTTATGA<br>GAGGCATGCCTGGTATAACTCG | Glushkevich et al., 2022 [65] | 350 | 97.6 |
| StCOX-F<br>StCOX-R | GGTCGGACATACCTGAAAC<br>CCAAAAGTATGAAAAGCTGGAG | Baebler et al., 2011 [59] | 350 | 97.3 |
| StEF-1α-F<br>StEF-1α-R | CTTGACGCTCTTGACCAGATT<br>GAAGACGGAGGGGTTTGTCT | Nicot et al., 2005 [58] | 350 | 98.7 |

Full references are provided in the main text. Primer concentrations giving the lowest threshold cycle ($C_t$) value were utilized in RT-PCR and are listed in the Table. E, efficiency of PCR amplification as calculated by CFX Manager Software.

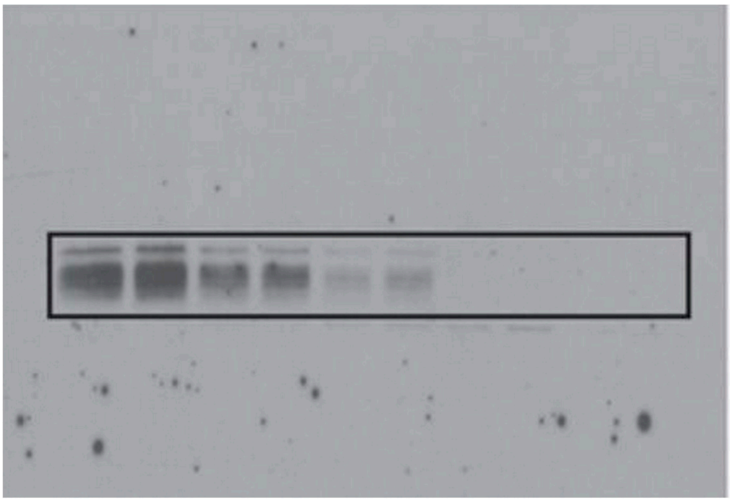

Figure S1: Uncropped Northern blot for Figure 2.
